# Chronic Thromboembolic Pulmonary Hypertension: A Narrative Review

**DOI:** 10.7759/cureus.104418

**Published:** 2026-02-27

**Authors:** Timea Jurth, Silvana Marasco

**Affiliations:** 1 Department of Cardiothoracic Surgery, The Prince Charles Hospital, Brisbane, AUS; 2 Department of Cardiothoracic Surgery, Alfred Health, Melbourne, AUS

**Keywords:** chronic thromboembolic pulmonary hypertension (cteph), dyspnea, group 4 pulmonary hypertension, pulmonary embolism (pe), pulmonary thromboendarterectomy for cteph

## Abstract

Chronic thromboembolic pulmonary hypertension (CTEPH) is a rare complication of acute pulmonary embolism (PE), which is chronically underdiagnosed. It is characterized by organized thromboembolic lesions within the pulmonary arteries, resulting in both obstruction of pulmonary arterial blood flow and pathologic small vessel remodeling. Patients present with dyspnea and decreased exercise tolerance; however, CTEPH patients are generally very comorbid, and these nonspecific symptoms often fail to initiate the diagnostic investigations. Late presentation results in poor outcomes with pulmonary hypertension and symptomatic right heart failure.

Surgical treatment with pulmonary thromboendarterectomy is the gold standard treatment for CTEPH; however, only proximal arterial disease is surgically accessible. Over a third of patients are ineligible for surgery due to a significant burden of distal disease or comorbidities. Other treatment modalities, such as balloon angioplasty and medical therapies, play an important role in the management of these patients.

Investigations for the diagnosis and treatment of CTEPH are extensive and require close multidisciplinary collaboration between medical, surgical, and imaging specialists. However, initiating the diagnostic investigations and referral process relies primarily on a high index of suspicion maintained by general practitioners, as early diagnosis is paramount to achieving successful outcomes in this cohort.

The objective of this narrative review is to summarize the current knowledge and understanding of CTEPH, including its pathogenesis, presentation, diagnosis, current treatments, and outcomes. We also highlight current areas of uncertainty and ongoing research. Improving awareness and understanding of CTEPH will ultimately lead to better outcomes in the treatment of this disease.

## Introduction and background

Why should a clinician consider chronic thromboembolic pulmonary hypertension (CTEPH) in a patient with persistent shortness of breath after a pulmonary embolism (PE)? This review addresses the diagnostic challenges and evolving management strategies.

CTEPH is a rare, progressive pulmonary vascular disease that develops as a late complication of acute PE [1]. Following an acute PE, restoration of normal pulmonary blood flow occurs through physiological or pharmacologically aided thrombolysis or physical clot removal (either percutaneously or surgically). Therapeutic anticoagulation is utilized to prevent further thrombosis or propagation of existing thrombus. In CTEPH, incomplete thrombolysis and often recurrent PE lead to organization and fibrosis of residual thrombotic material, which results in obstruction of pulmonary arteries and secondary small vessel remodeling. The latter refers to pathological changes in the arterioles and capillaries of the lung, resulting in increased blood pressure and resistance in the pulmonary circulation. Both obstruction and small vessel remodeling contribute to pulmonary hypertension and ultimately right ventricular failure as the final pathological endpoint [2].

Long-term prognosis has significantly improved since the 1980s, coinciding with improvements in all modes of treatment and in the implementation of multimodal treatment. A recent study has shown five-year survival rates have improved from 68% to 93% over the past 45 years [3]. 

CTEPH is classified as a Group IV pulmonary hypertensive disease by the World Health Organization (WHO) guidelines, distinct from other causes of pulmonary hypertension such as lung disease or left heart failure [4]. Surgical treatment with pulmonary thromboendarterectomy (PTE) offers a potentially curative treatment for CTEPH [5], whereby organized thromboembolic material is physically removed from the pulmonary arteries down to the subsegmental level.

Outcomes following PTE are often unpredictable, with residual high pulmonary arterial pressures, high pulmonary vascular resistance, and persisting symptoms observed not infrequently despite successful resection of thromboembolic material [6]. Other treatment modalities are balloon pulmonary angioplasty (BPA) and medications, which are also used as an adjunct to surgical treatment [7]. Careful patient selection is therefore paramount in the successful treatment of this debilitating disease. 

This narrative review summarizes current evidence and practices in CTEPH, with an emphasis on diagnostic pathways, multidisciplinary management, and emerging therapies, to aid clinicians in recognizing and treating this underdiagnosed condition.

## Review

Methods

For this review, a literature search was conducted in the PubMed database from inception to January 2026. The following terms were searched: CTEPH, chronic thromboembolic pulmonary hypertension, chronic thromboembolic disease, pulmonary thromboendarterectomy, pulmonary endarterectomy, and balloon pulmonary angioplasty. Inclusion criteria were English-language, human studies, peer-reviewed articles, clinical trials, and guidelines, whilst exclusion criteria were case reports and non-English abstracts. Given the narrative nature of this review, a formal risk-of-bias assessment was not performed, but priority was given to international guideline recommendations, larger cohort studies, and research from high-volume expert CTEPH centers. Approximately 300 abstracts were screened, with chosen articles selected based on relevance and author consensus. All referenced articles were read in full by the lead author. 

History and epidemiology of CTEPH

CTEPH was first described as early as 1929 [8], but it wasn’t until 1963 that the first case report of surgical management with PTE appeared in the literature [9]. It is interesting to note that PTE preceded heart-lung transplantation in the treatment of CTEPH by nearly 20 years [10].

The incidence of CTEPH has not been clearly established, with estimates in the literature ranging between 0.1% and 9.1% within two years of an acute PE [6,7,11,12]. Several factors contribute to this variation, including referral and selection bias. Additionally, inherent difficulties in differentiating between the symptoms of acute PE and those of pre-existing CTEPH result in an unintentional mix of incident and prevalent cases even in prospective epidemiologic studies [1,7,13].

Epidemiological research has focused on CTEPH as a complication of acute PE; however, several studies suggest that CTEPH pathology may exist in the absence of a known history of PE or venous thromboembolism (VTE) in 25% to 50% of patients [14-16]. This group is excluded from prospective epidemiologic analyses of PE registries, thereby significantly underestimating the burden of disease. Nevertheless, recent studies suggest the crude full incidence of CTEPH in the USA and Europe is about four cases per 100,000 population per year, with a diagnosed incidence of about five cases per 1,000,000 population per year [7,17]. In contrast, the incidence of acute PE is about one in 1000 per population year [18]. This indicates that CTEPH is dramatically underdiagnosed (and undertreated), an issue that has been described since the 1950s [19]. The median age of diagnosis is 63 years, with equal distribution between genders [14].

Pathophysiology

**Figure 1:**
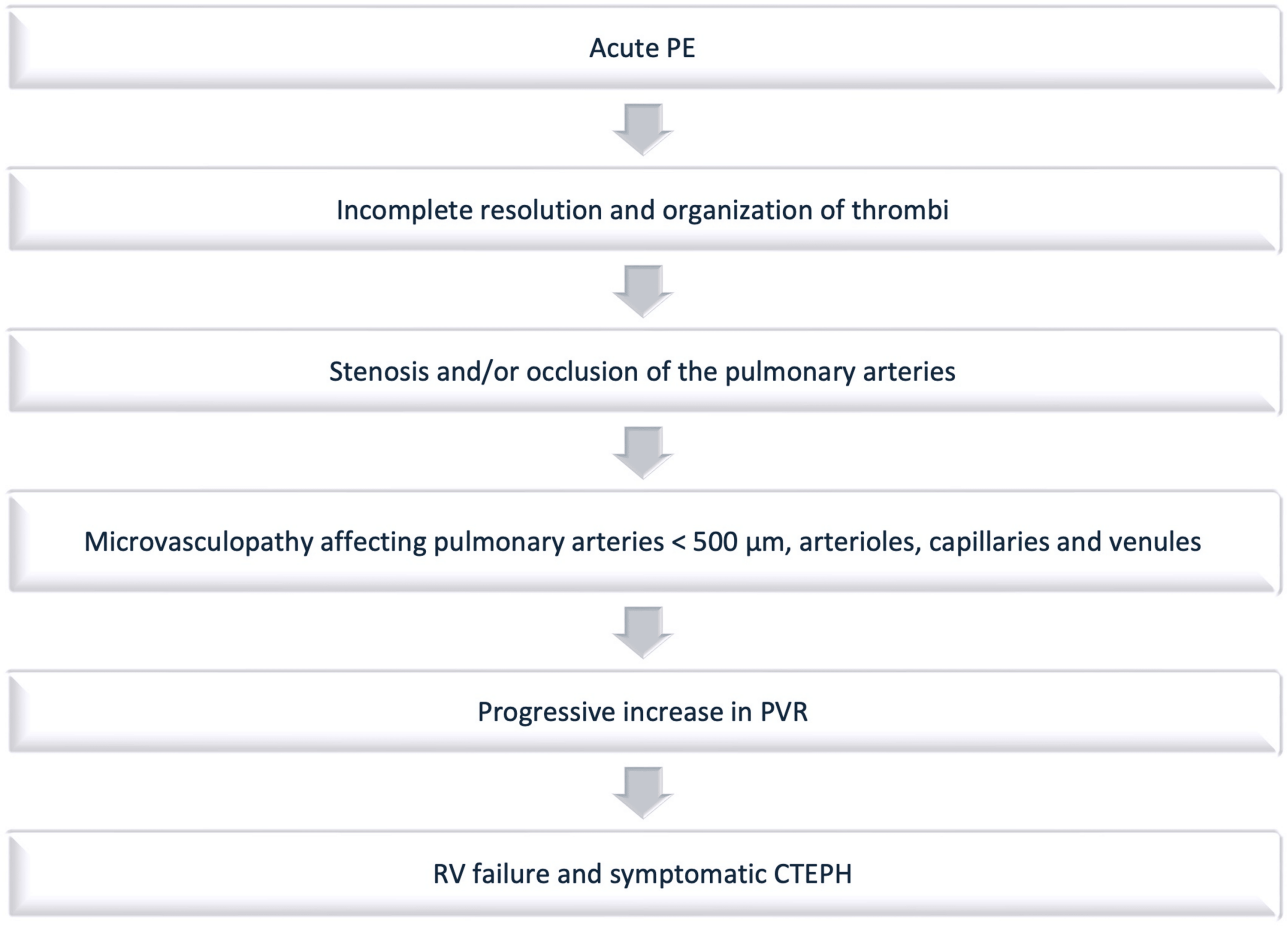
The pathophysiology of CTEPH showing the natural progression of disease PE: pulmonary embolism; PVR: pulmonary vascular resistance; RV: right ventricle; CTEPH: chronic thromboembolic pulmonary hypertension Source:  [6,22]

The WHO classifies CTEPH as a Group IV pulmonary hypertensive disease, distinct from other causes of pulmonary hypertension such as pulmonary arterial hypertension (Group I), pulmonary hypertension due to left heart disease (Group II) or lung disease (Group III), and unknown causes (Group V) [4]. The mechanism of pulmonary hypertension in CTEPH is multifactorial, characterized by several distinct entities including chronic mechanical obstruction of the pulmonary arteries by organized thrombotic material, secondary microvasculopathy, and vascular remodeling, all of which contribute to increased pulmonary vascular resistance (PVR), chronic right ventricular (RV) afterload, and ultimately RV failure if left untreated [7] (Figure 1). Impaired RV function presents a significant challenge in the management of these patients. The hypertrophied, dilated RV leads to annular dilatation and functional tricuspid regurgitation, ventricular interdependence, and leftward shift of the interventricular septum, thereby impairing left ventricular diastolic filling and reducing cardiac output. This further increases RV preload, resulting in a cycle of dysfunction [20]. Furthermore, RV impairment has been found to be the main determinant of symptoms and prognosis in this cohort [21]. 

Pulmonary Arterial Obstruction

In CTEPH, thrombus resolution following PE is impaired for reasons that are still poorly understood [23]. Chronic obstruction or partial obstruction by unresolved organized fibrotic material can affect the pulmonary arterial tree down to the level of the distal pulmonary arteries [7]. Obstructive lesions can partially recanalize, forming multiple secondary lumina, or reorganize into rings, bands, and webs, which are characteristic lesions seen on pulmonary angiography [12]. 

The physical obstruction of the pulmonary arterial circulation increases RV afterload and PVR, and the thrombotic material adherent to the vessel wall decreases vessel compliance. In addition, large elastic pulmonary arteries under the strain of long-term elevated pulmonary arterial pressures have been shown to develop atherosclerotic lesions resembling the aortic wall pathology found in systemic hypertension [24]. These lesions further contribute to vessel wall stiffness and decreased pulmonary compliance. 

Histologically, thromboembolic lesions are predominantly comprised of collagen, in addition to elastin and a range of inflammatory cells [25]. A proposed mechanism to explain the development of the thromboembolic lesions involves the activation, differentiation, and migration of smooth muscle cells in the tunica media into α-SMA-positive cells, which then deposit collagen and elastin fibers in the intimal layer [26].

Numerous studies suggest that chronic inflammation and impaired angiogenesis play a key role in the pathogenesis of CTEPH. Compared to healthy controls, CTEPH patients show higher levels of CRP, as well as interleukin (IL)-10, IL-6, IL-8, interferon-γ-induced protein (IP)-10, macrophage inflammatory protein-1α, monocyte chemotactic protein-1, and matrix metalloproteinase (MMP)-9 [25,26]. 

Secondary Microvasculopathy

The mechanism behind small vessel vasculopathy in CTEPH is incompletely understood but involves vasculature downstream of both obstructed and non-obstructed vessels [6]. Small vessel vasculopathy in CTEPH was first described by Moser and Bioor over 30 years ago from tissue samples taken during lung biopsy or at autopsy [27]. These specimens showed lesions in the vessel wall of pulmonary arteries 50-500 µm in diameter and were limited to areas without proximal occlusion. The pathological changes were thought to be a result of shunted blood from occluded areas to non-occluded areas, resulting in increased flows, pressure, and vessel wall shear stress. These observations have since been reported in more recent literature together with changes of intimal thickening and fibrosis, medial hypertrophy, and plexiform lesions present in the small muscular arteries and arterioles [13,28].

Vasculopathy observed downstream of occluded areas has a different proposed mechanism. Downstream lesions involve pre-capillary arteries, capillaries, and post-capillary venules, leading to changes resembling hemangiomatoses and veno-occlusive disease in the microcirculation. The mechanism is thought to be secondary to hypertrophy of the bronchial arteries and development of bronchopulmonary anastomoses. These changes have been observed on both the arterial and venous sides of the capillaries in animal models, resulting in the transmission of systemic blood pressures to the pulmonary circulation [29].

A significant consequence in the preoperative evaluation of CTEPH is that the relative contribution of secondary microvasculopathy to pulmonary hypertension, PVR, and gas exchange is often unpredictable, especially in the context of a large burden of proximal thromboembolic material. In these patients, surgical treatment with PTE has limited success despite adequate resection [12].

Genetics of CTEPH

Separating the genetic susceptibility of deep vein thrombosis (DVT)/PE from CTEPH is challenging; however, a large genome-wide association study comparing patients with DVT, PE, idiopathic pulmonary arterial hypertension, and CTEPH showed shared heritable genetic architecture between DVT, PE, and CTEPH. Genetic associations were found at the ABO, FGG, F11, MYH7B, and HLA-DRA loci [30]. Importantly, these associations were related, although distinct from PE and DVT. 

Other research has shown variants in the STAB2 gene, which encodes stabilin-2, have been found to be significantly over-represented in patients with CTEPH. STAB2 variants showed elevated levels of plasma von Willebrand factor and factor VIII, indicating a specific role in the failure of thrombus resolution [31,32].

Risk factors

Risk factors associated with the development of CTEPH include acute embolic events, thrombophilia with its associated conditions, and a range of other pathologies [33,34]. Both the presence of previous episodes of PE and VTE and the extent of the initial perfusion defect are significant risk factors for CTEPH [1]. Other clinical conditions associated with CTEPH include splenectomy, inflammatory bowel disease, osteomyelitis, myeloproliferative syndromes, neoplastic pathology, infected lines and devices such as pacemakers or venous catheters, and ventricle-atrium shunting in the treatment of hydrocephalus [6,34-37]. The latter is thought to be a consequence of infection, causing delayed thrombus resolution, up-regulating transforming growth factor-β, and connective tissue growth factor thrombus formation [38]. Hypothyroidism with hormone replacement therapy is also a risk factor in its association with increased plasma levels of von Willebrand factor [39].

Thrombophilia has been extensively studied in the context of CTEPH. Lupus anticoagulant/antiphospholipid antibodies are present in 10% to 20% of patients, and elevated plasma factor VIII is found in 25% of patients with CTEPH [35,40]. Interestingly, no correlation has been found between CTEPH and deficiencies in antithrombin III, protein C, protein S, or the factor V Leiden mutation despite routine screening for these disorders in CTEPH patients in most centers [7,23,41].

Presentation and diagnosis

CTEPH presents most commonly with persistent dyspnea and decreased exercise tolerance following an acute PE or DVT; however, a preceding thromboembolic event is absent in up to 50% of cases in the literature [6,14-16]. The timing between the index thromboembolic event and the onset of CTEPH has proved difficult to determine for several reasons. Symptoms and signs in early CTEPH may be minimal or nonspecific [12]. In addition, CTEPH is commonly associated with comorbidities such as morbid obesity, obstructive sleep apnea, and chronic pain, which can mask symptoms attributable to CTEPH, further delaying diagnosis. 

General practitioners are in a key position to initiate appropriate investigations and referrals to CTEPH specialists. However, timely diagnosis relies on awareness of this rare disease and maintaining a high index of suspicion in patients with symptoms and risk factors, especially if symptoms persist following three months of anticoagulation. Median time between symptom onset and diagnosis is 14 months in expert centers [23]. Delays in diagnosis result in late presentation with advanced disease and symptomatic right heart failure. Nevertheless, although algorithms for the follow-up of patients following acute PE have been established, routine screening for CTEPH after PE, even in high-risk patients, is not supported in CTEPH guidelines [6].

The diagnostic criterion for CTEPH is as follows: 1. Ventilation/perfusion (V/Q) scanning showing persistent mismatched perfusion defects. 2. Right heart catheterization (RHC) showing an elevated mean pulmonary artery pressure (PAP) >20 mmHg, pulmonary capillary wedge pressure (PCWP) ≤15 mmHg, and PVR >2 Wood units (WU). 3. Diagnosis is only confirmed following three months of anticoagulation to discriminate between CTEPH and subacute PE [6].

Although the diagnostic investigations are V/Q scanning and RHC, in practice, the initial investigations are usually computed tomography pulmonary angiography (CTPA) and transthoracic echocardiogram (TTE) in patients presenting with persistent dyspnea. Although not diagnostic, these tests can be suggestive of CTEPH whilst ruling out many differential diagnoses presenting with the same non-specific symptoms.

Chronic thromboembolic disease (CTED)

CTED is distinct from CTEPH. It is characterized by persistent thromboembolic occlusions with symptoms in the absence of pulmonary hypertension or elevated PVR [6]. CTED is not necessarily a precursor to CTEPH, and patients generally do not develop right heart failure. PTE in the treatment of CTED has shown significant symptomatic benefit and improvement in quality of life in a highly selective cohort of patients [42].

Investigations and work-up for treatment

**Figure 2:**
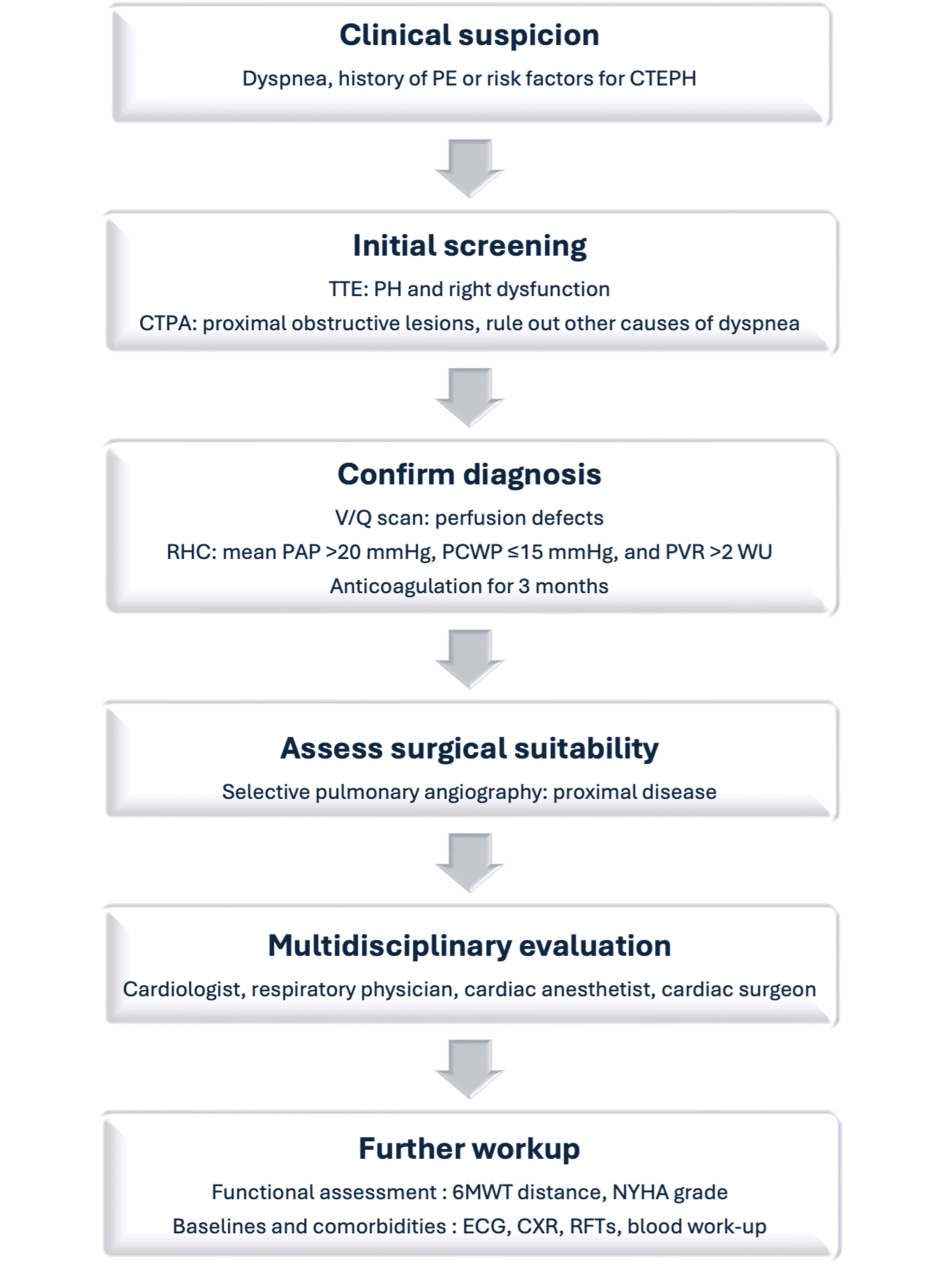
A diagnostic algorithm for CTEPH PE: pulmonary embolism; CTEPH: chronic thromboembolic pulmonary hypertension; TTE: transthoracic echocardiogram; PH: pulmonary hypertension; CTPA: computed tomography pulmonary angiogram; V/Q: ventilation/perfusion; RHC: right heart catheterization; PAP: pulmonary artery pressure; PCWP: pulmonary capillary wedge pressure; PVR: pulmonary vascular resistance; WU: Wood units; 6MWT: six-minute walk test; ECG: electrocardiogram; CXR: chest X-ray; RFTs: respiratory function tests Source: [6,43]

Patients investigated for CTEPH undergo numerous investigations as they progress through the diagnostic pathway (Figure 2), instigated by a range of clinicians, which highlights the multidisciplinary collaboration involved in the diagnosis and management of CTEPH. Treatment decisions are made following anatomical, hemodynamic, and symptomatic assessment to characterize the distribution of the disease (proximal and/or distal disease), severity of disease, suitability of surgical intervention, evaluation of significant comorbidities, and to establish baseline values. 

Transthoracic Echocardiogram

TTE is recommended as the first line in the investigation of CTEPH in the establishment and assessment of right heart dysfunction [6]. TTE is also necessary in the evaluation of left heart pathology and in the detection of concomitant valvular and congenital abnormalities in patients being considered for surgical management.

Ventilation/Perfusion Scintigraphy

Despite recent advances in CT and magnetic resonance imaging (MRI) imaging modalities, V/Q scanning remains the primary diagnostic investigation [7]. V/Q scanning in CTEPH has a sensitivity of 90% to 100% and a specificity of 94% to 100%, which far exceeds the sensitivity of CTPA (sensitivity 51%, specificity 97%), particularly in inexperienced centers [6,44]. A diagnosis of CTEPH can therefore be confidently ruled out following a normal or low-probability V/Q scan.

Computed Tomography Pulmonary Angiogram

CTPA is used widely in the diagnosis of acute PE; however, it cannot be used alone in the diagnosis of CTEPH [6]. Features suggestive of CTEPH include dilated pulmonary arteries with intravascular webs or bands, including mural thrombus, arterial occlusion, presence of large bronchial arterial collaterals, RV hypertrophy, and the characteristic lesions of hypoperfusion (hyper-transparency) that constitute the “mosaic pattern” [45]. However, the mosaic pattern is also present in 12% of patients with idiopathic pulmonary arterial hypertension [46]. The primary utility of CTPA is in the assessment of concomitant lung pathology such as emphysema, bronchial or interstitial lung disease, as well as vascular malformations and thoracic wall deformities [12]. CTPA is also useful in detecting interval change or recurrent PE in patients following an initial diagnosis of acute PE.

Right Heart Catheterization

RHC plays a central role in the diagnosis of CTEPH, in the assessment of CTEPH severity, and in the monitoring of postoperative outcomes [7]. A balloon-tipped catheter is inserted into a large peripheral vein, which is then sailed into the heart, sequentially obtaining direct pressure measurements from the right atrium, right ventricle, and pulmonary artery. The catheter balloon can be inflated in a distal pulmonary artery to obtain a ‘wedge’ pressure, a reading that is commonly used as a surrogate for left atrial pressure. Other measurements obtained during RHC include cardiac output and mixed venous oxygen saturation.

Despite the dependence of these measurements on the evaluation and management of CTEPH, it is important to acknowledge the limitations of RHC. Values obtained from the RHC are integrated with hemodynamic values to yield an enormous range of extrapolated data, several of which include assumptions based on “normal” physiological scenarios. An example of this is seen in PCWP as an indirect measurement of left atrial pressure. This value assumes normal lung parenchyma and physiology between the wedged pulmonary artery and the left atrium, which is certainly incorrect in the setting of CTEPH [28].

Selective Pulmonary Angiography

Selective pulmonary angiography plays a key role in the evaluation of surgical suitability and operative planning, specifically in assessing the location and burden of proximal disease amenable to resection. Findings of pathognomonic features such as ring-like stenoses, webs, bands, and pouches further confirm the diagnosis of CTEPH [47,48].

Six-Minute Walk Test (6MWT)

The 6MWT is a submaximal exercise test used routinely as an objective assessment of functional capacity both before and after treatment. Performed according to the guidelines of the American Thoracic Society [49], patients are instructed to walk a measured length of corridor at their own pace and cover as much distance as possible over six minutes. Parameters recorded include the distance covered, the number of stops taken during the walk, the lowest oxygen saturation level, and the use of supplemental oxygen. The test is reproducible, requires no specialized equipment, and accurately reflects normal physical activity undertaken by all except the most impaired patients. Furthermore, the 6MWT distance has been found to correlate with both the clinical and hemodynamic severity of disease, including New York Heart Association (NYHA) functional class and degree of pulmonary hypertension [50].

The NYHA Functional Classification

The NYHA classification grades heart failure symptoms as a reflection of functional capacity. Class I describes no symptoms or limitations to physical activity, Class II describes mild symptoms or slight limitation during ordinary activity, Class III describes marked limitation in activity due to symptoms, even during less-than-ordinary activity, and Class IV describes severe limitations with symptoms present at rest [51]. The NYHA classification is widely used in the functional assessment of CTEPH preoperatively as a measure of disease severity and also following treatment as a measure of outcome [7].

The main limitation of measures of functional capacity is that common comorbidities in this cohort, such as chronic obstructive pulmonary disease, obstructive sleep apnea, osteoarthritis, morbid obesity, and chronic pain, can significantly affect mobility and exercise capacity, thereby limiting the anticipated improvement that could be conferred by successful treatment.

Management of CTEPH

Treatment modalities in CTEPH include PTE, BPA, and pulmonary vasodilator therapy, including various combinations as multimodal therapy. PTE offers a potentially curative outcome and remains the gold standard in the management of CTEPH [7]. However, 36% of patients are considered ineligible for surgical management due to a range of factors, including distal pulmonary artery obstructions, imbalance between severity of pulmonary hypertension and morphologic lesion, PVR >19 WU, and comorbidities [7,52]. Medical therapies are not limited to inoperable CTEPH but also play a role in recurrent or residual disease following PTE [53]. This latter group can include up to 35% of patients post PTE [53,54] and therefore, understanding the applications and limitations of medical therapies is crucial regardless of the treatment strategy. 

Pharmacological Treatment 

Pharmacological treatment includes anticoagulant therapy, pulmonary vasodilators, and other therapies aimed at symptom management and congestive heart failure (diuretics and supplemental oxygen). Lifelong anticoagulation is recommended to prevent further pulmonary thromboembolic events [12]. Vitamin K antagonists (VKAs) are preferred in most centers, although novel direct oral anticoagulants (DOACs) are increasingly utilized in this setting; however, there are currently no randomized controlled trials comparing outcomes of VKAs to DOACs in this cohort [6].

Pulmonary hypertension is associated with endothelial dysfunction, impaired synthesis of nitric oxide, and insufficient stimulation of the nitric oxide-soluble guanylate cyclase-cyclic guanosine (NO-sGC-cGMP) pathway, resulting in vasoconstriction, proliferation, fibrosis, and inflammation [53]. Pulmonary antihypertensive therapy used in CTEPH, therefore, acts on the NO-sGC-cGMP pathway. Current guidelines recommend only riociguat (an sCG stimulator); however, many centers use sildenafil off-label (prevents the breakdown of cGMP through selective phosphodiesterase-5 (PDE5) inhibition) and bosentan (an endothelin receptor antagonist inhibiting vasoconstriction) [6]. Additionally, nitric oxide, an endogenous vasodilator, is often utilized in the immediate postoperative setting [55].

Balloon Pulmonary Angioplasty

BPA is a percutaneous catheter-based treatment option in the setting of inoperable CTEPH or in the setting of recurrent or residual disease [14,56]. Vascular access is obtained via the internal jugular or femoral vein, and selective pulmonary angiography is used to identify the lesions. A balloon-tipped catheter is then dilated within the target lesions to restore pulmonary blood flow. Lesions amenable to BPA are limited to webs, nets, and fissures, whilst completely occluded vessels cannot be treated. An average of 4.2 sessions is required to show hemodynamic improvement. BPA-related lung injury is as high as 60%; however, associated mortality is relatively low at 0-3.4% [57-59].

Multimodal Treatment 

As imaging, interventions, and medical therapies develop, focus is increasing on multimodal approaches to the treatment of CTEPH. Patients often demonstrate a heterogeneous pattern of disease, with surgically accessible lesions in some areas and inoperable lesions in others. These patients may benefit from a combination of surgery, BPA, and medical therapy, although there is currently limited data on outcomes in these types of patients [6]. 

A randomized controlled trial comparing riociguat to BPA in the treatment of inoperable CTEPH found that BPA was more effective than riociguat at lowering PVR at 26 weeks but similar at 52 weeks following treatment. Treatment-related complications were fewer in the riociguat group. Importantly, patients pretreated with riociguat before undergoing BPA showed fewer BPA-related serious adverse events, suggesting potential benefits of a multimodal approach in this cohort [60].

There is currently no evidence for medical therapy as a “bridge to PTE," and concerns have been raised that it would potentially delay definitive treatment without conferring any clinical benefit [58,61]. 

Pulmonary thromboendarterectomy patient selection

Patients with CTEPH are a comorbid group with an over-representation of clotting disorders, morbid obesity, concurrent respiratory disease, and systemic hypertension. This contributes to surgical and anesthetic risk in an already technically demanding operation. Careful patient selection is, therefore, paramount and involves multidisciplinary collaboration between respiratory cardiologists, respiratory physicians, radiologists, cardiac anesthetists, and surgeons with subspecialty expertise and experience in CTEPH [7].

Importantly, patients suitable for PTE must have a significant disease burden within the proximal pulmonary arteries. Distal arterial disease is not surgically accessible, and therefore, patients with a predominantly distal pattern of disease will not benefit from surgical intervention. Age in itself is not a contraindication to surgical treatment [7,62].

Pulmonary thromboendarterectomy operative technique 

**Figure 3:**
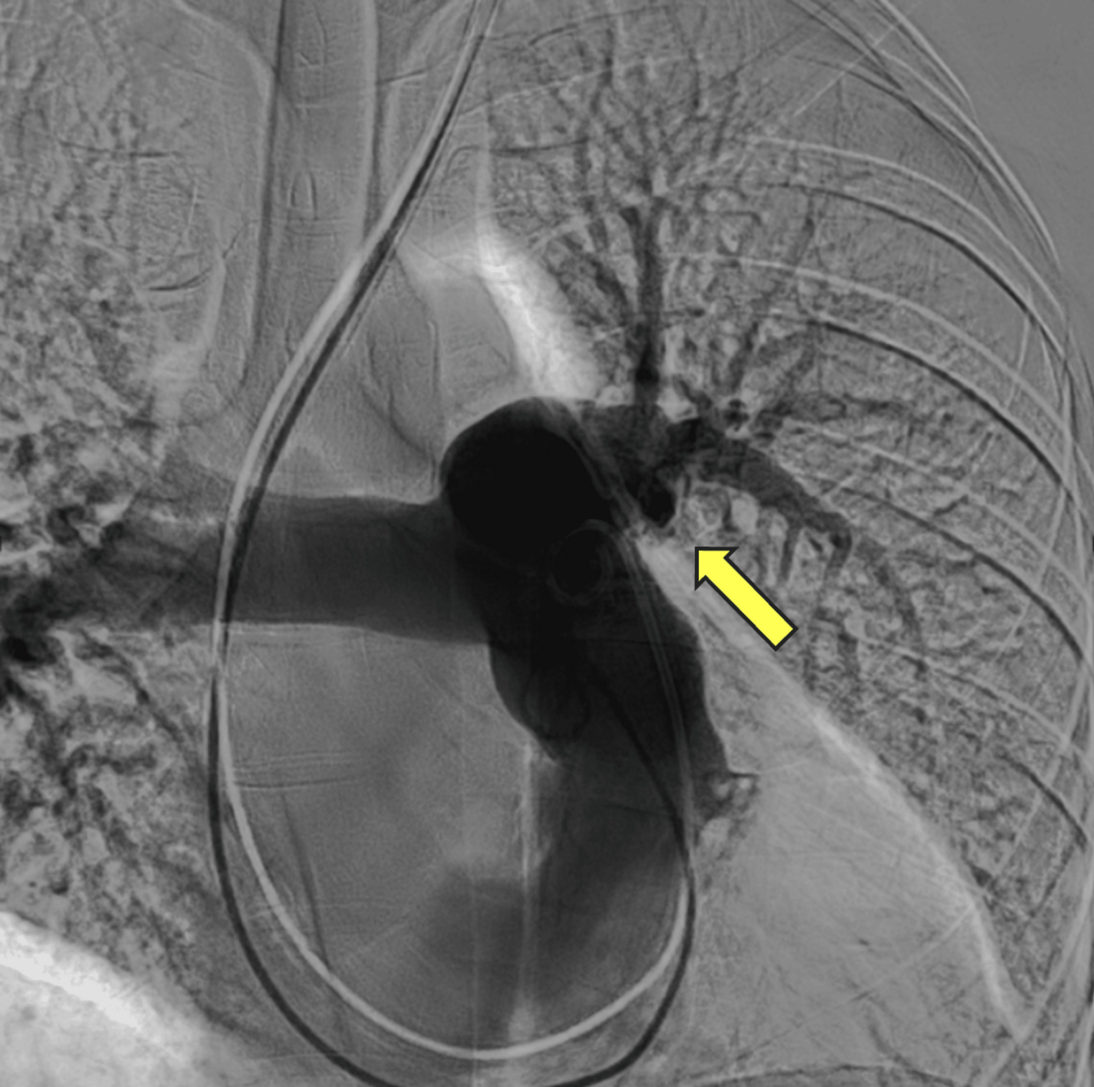
Preoperative selective pulmonary angiogram shows complete occlusion of the lower left lobe pulmonary vasculature (arrow). Note: This image has been captured by the authors.

**Figure 4:**
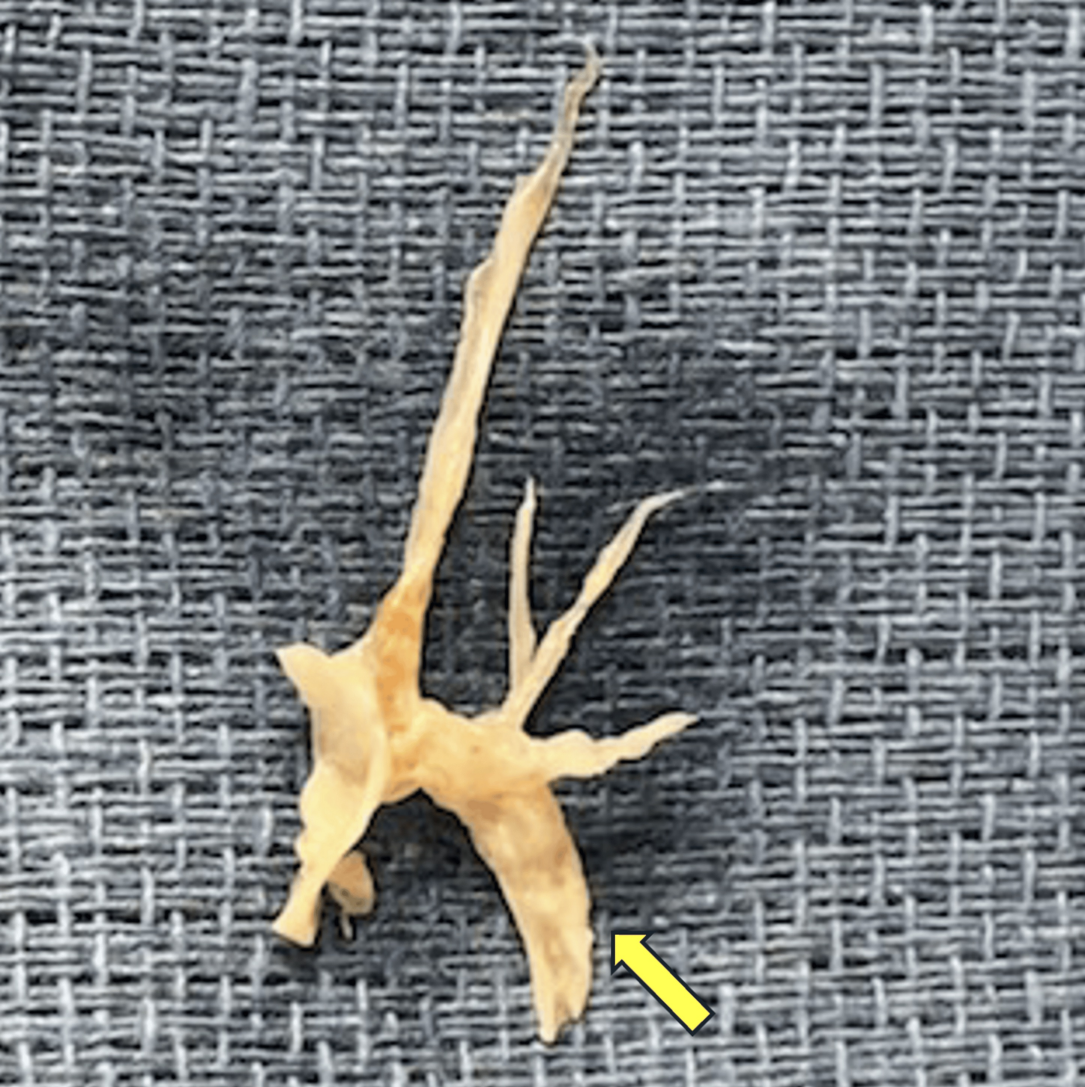
The corresponding intraoperative specimen of thromboembolic material removed from the left pulmonary artery with a large lesion taken from the left lower lobe artery (arrow). Note: This image has been captured by the authors.

The surgical technique for PTE is well established in the literature [62,63]. Following a median sternotomy, cardiopulmonary bypass is established, and systemic cooling is commenced. Circulatory arrest is initiated when the patient reaches 20°C. An incision is then made sequentially over the proximal pulmonary arteries. Thromboembolic material is pale yellow, densely adherent to the vessel wall, and can be difficult to distinguish at its proximal origin. A plane is established within the tunica media layer, and the thromboembolic material is gently dissected from the vessel wall until the “tails” of the lesion are freed distally at the subsegmental level. Selective pulmonary angiography performed preoperatively can provide a “road map” identifying occluded vessels to help guide the anatomical extent of the resection (Figures 3, 4). Establishing the correct dissection plane is crucial to the success of the surgery. Developing an incorrect plane can result in the incomplete removal of thromboembolic material, premature breakage of the tails, leaving residual distal material, and pulmonary arterial perforation. 

Outcomes following PTE

Hemodynamics following PTE improve rapidly to normal or near-normal in the majority of patients, with an average decrease in PVR and mean PAP by 65% and 44%, respectively. The average 6MWT distance increases from 362 to 459 meters, together with a decrease in the NYHA functional class, with most patients progressing from NYHA class III/IV to class I/II [52].

Current data show CTEPH treatment is associated with a three-year survival of 94% and 92% in patients undergoing PTE and BPA, respectively, and 71% in patients treated with medical therapies alone [56].

Future directions 

As CTEPH management continues to evolve, the importance of a multidisciplinary team approach is increasingly invaluable, a concept that is emphasized in the most recent guidelines [6] and championed in expert centers [64]. Whilst lacking in scientific evidence, multidisciplinary approaches with subspecialty expertise enhance the utilization of multimodal therapy, thereby expanding treatment options for CTEPH patients. 

Despite therapeutic advances, only riociguat is currently recommended in the treatment of CTEPH [6]. Medications such as sildenafil and bosentan are currently used off-label, and whilst they have been shown to confer benefit, robust randomized controlled trials are needed to expand this field [65]. 

Sotatercept is a novel biologic that inhibits activin signaling by acting as a ligand trap for transforming growth factor-β. Although not specific to CTEPH, a recent meta-analysis of sotatercept used in pulmonary arterial hypertension has shown significant improvement in hemodynamics and functional capacity, potentially representing a new treatment option for CTEPH patients [66,67]. 

Research investigating the genetic basis for the development or progression of CTEPH is still in its infancy. Nevertheless, variations in genes related to coagulation, fibrinolysis, and platelet disorders could serve as the basis of future research and potentially therapeutic targets [31,32,68].

## Conclusions

CTEPH is a potentially curable disease, but presents a diagnostic challenge due to its rarity and lack of awareness, and nonspecific symptoms at presentation. Despite exciting developments in therapies, delays in diagnosis represent the greatest barrier to better outcomes. CTEPH should always be considered in the setting of persistent dyspnoea following an acute PE, prompting early investigation and referral to CTEPH specialists. Initial investigations are CTPA and TTE, which, although not diagnostic, are more readily available and can be highly suggestive of CTEPH.

Current management aims to address the different aspects of disease, namely, pulmonary arterial obstructive disease and microvasculopathy. Multidisciplinary team approaches and tailored multimodal therapy will improve diagnosis, management, and outcomes of this debilitating disease.
